# Antimicrobial Peptides As Biologic and Immunotherapeutic Agents against Cancer: A Comprehensive Overview

**DOI:** 10.3389/fimmu.2017.01320

**Published:** 2017-10-13

**Authors:** Raheleh Roudi, Nicholas L. Syn, Maryam Roudbary

**Affiliations:** ^1^Oncopathology Research Center, Iran University of Medical Sciences, Tehran, Iran; ^2^Department of Haematology-Oncology, National University Cancer Institute, Singapore, Singapore; ^3^Yong Loo Lin School of Medicine, National University of Singapore, Singapore, Singapore; ^4^Department of Medical Mycology and Parasitology, School of Medicine, Iran University of Medical Sciences, Tehran, Iran

**Keywords:** antimicrobial peptides, cancer immunotherapy, biopharmaceuticals, anticancer drugs, microbiome

## Abstract

Antimicrobial peptides (AMPs) are a pervasive and evolutionarily ancient component of innate host defense which is present in virtually all classes of life. In recent years, evidence has accumulated that parallel or *de novo* mechanisms by which AMPs curb infectious pathologies are also effective at restraining cancer cell proliferation and dissemination, and have consequently stimulated significant interest in their deployment as novel biologic and immunotherapeutic agents against human malignancies. In this review, we explicate the biochemical underpinnings of their tumor-selectivity, and discuss results of recent clinical trials (outside of oncologic indications) which substantiate their safety and tolerability profiles. Next, we present evidence for their preclinical antitumor activity, systematically organized by the major and minor classes of natural AMPs. Finally, we discuss the barriers to their clinical implementation and envision directions for further development.

## Introduction

Cancer continues to take a toll on global public health systems, accounting for an estimated 8.7 million deaths annually (1, 2). In 2015, 17.5 million incident cases were diagnosed worldwide, and this is projected to spiral to 22.2 million by 2030 (1–3). Despite tremendous progress in reducing mortality rates from cancer and transformative shifts in therapeutic paradigms over the past few years, the development of novel therapeutic approaches remains an urgent priority, particularly in the setting of advanced, treatment-refractory malignancies.

**Figure 1:**
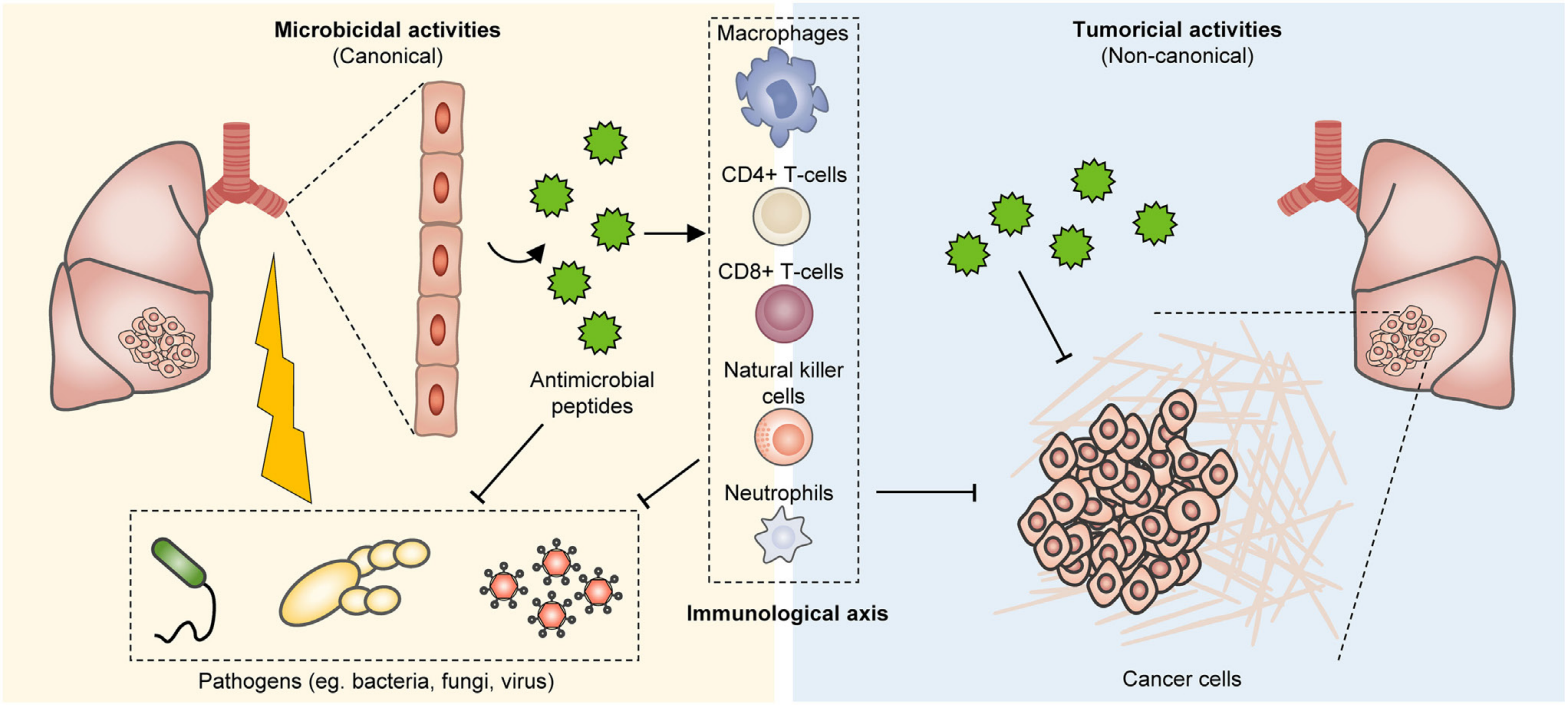
Bioproduction of antimicrobial peptides (AMPs) and their tumor suppressive effects. In humans, AMPs are present at various tissues, including the epithelium of the skin, respiratory system, and gastrointestinal tract, as well as the immune system. These peptides can effectively impinge on a broad spectrum of microorganisms including fungi, bacteria, and viruses. However, they also restrain tumor growth and their immunostimulatory properties further co-opt anticancer immunity for enhanced tumor eradication.

Owing to the clinical success of cancer immunotherapy, as exemplified by the broad efficacy of immune checkpoint inhibitors (e.g., pembrolizumab, ipilimumab, atezolizumab) across multiple tumor histologies, there has been renewed interest in the development of immunomodulatory strategies in oncology treatment (4). Antimicrobial peptides (AMPs) are structurally diverse, critical effector molecules of the innate immunity which rapidly act to inactivate invading microorganisms, especially at mucosal surfaces and epithelial barriers. Despite most translational studies involving AMPs being oriented toward their development as antibacterial and antifungal biopharmaceuticals, pioneering research has led to the identification of numerous AMPs with promising anticancer properties (5–14) (Figure 1). These include, but are not limited to various α-helical peptides, β-Sheet peptides, linear peptides, hybrid, and synthetic peptides (5, 15–18).

## Bioproduction, Physicochemical Properties, and Functions of AMPs

Antimicrobial peptides are small molecular weight oligopeptides, that is, they generally comprise 5–40 amino acid residues, with few exceptions. Both eukaryotes and prokaryotes are capable of producing these peptides. In humans, AMPs are present at various tissues, including the epithelium of the skin, respiratory system, and gastrointestinal tract, as well as the immune system (Figure 1). Depending on the site of synthesis, AMPs are broadly classified as non-ribosomal peptides (NRAMPs) if they are synthesized in the cytosol of bacteria and fungi, or ribosomal peptides (RAMPs) when they are synthesized in the ribosomes of both prokaryotes and eukaryotes (19, 20). AMPs are structurally heterogeneous and may assume linear (with amphipathic and hydrophobic α-helical residues [~30% or more]), β-sheet, cyclic, lipo, macrocyclic, or α-helical rod conformations (21, 22). It is worth noting that this diversity arises in part from post-translation modifications including glycosylation, phosphorylation, and amidation (23–27). AMPs are mostly cationic with a net charge at neutral pH ranging between +2 and +9 due to the presence of positively charged residues (typically, lysine [Lys] and arginine [Arg]), which endow these peptides with the ability to engage with and disrupt microorganismal membranes.

**Figure 2:**
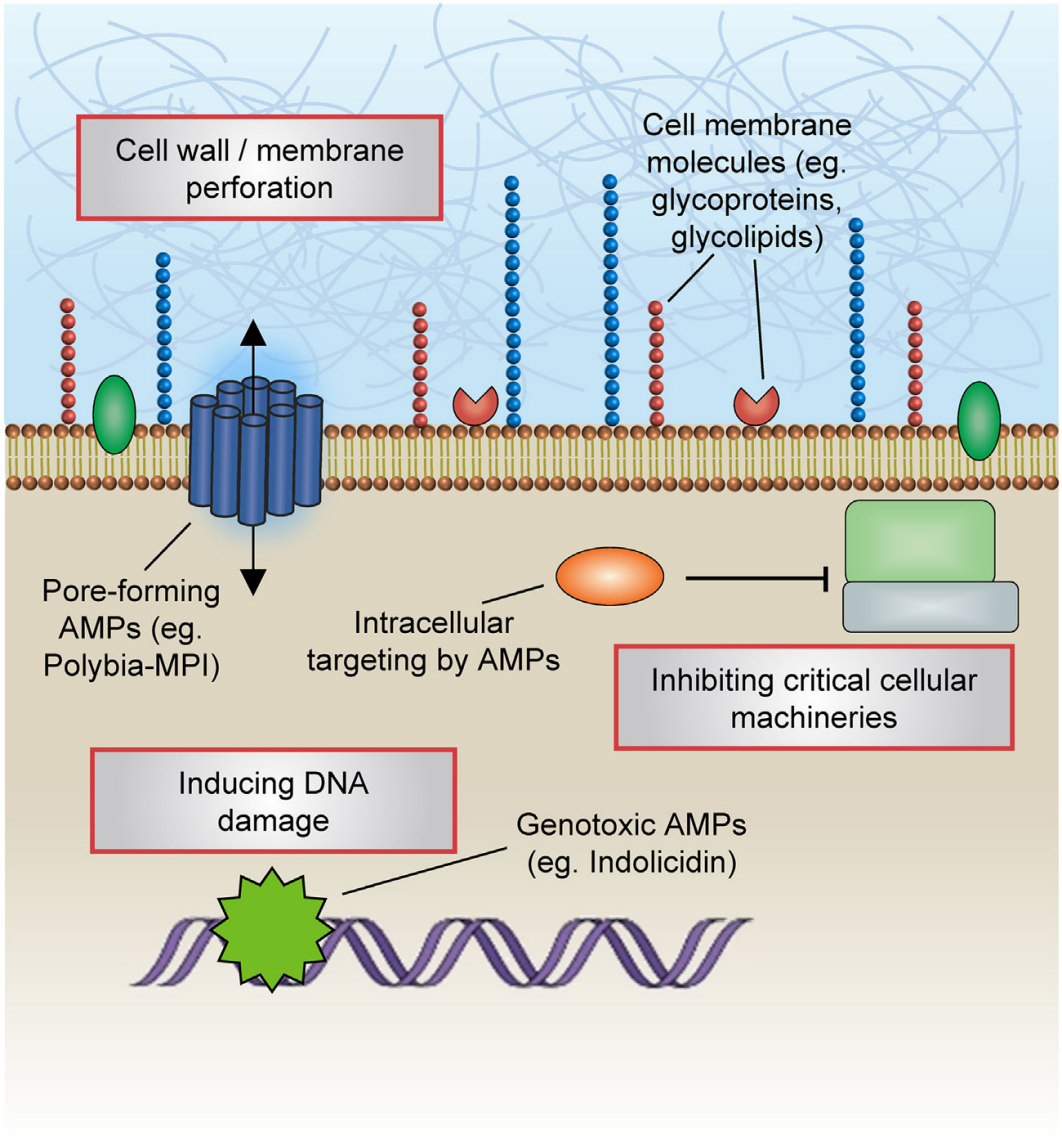
Mechanisms of antimicrobial actions. Classical mechanisms which mediate the antibiotic actions of antimicrobial peptides (AMPs) include their penetration of the plasma membrane or cell wall, thus resulting in lysis or disruption of ionic gradients; binding to and damaging nucleic acids (e.g., DNA); and blockade of enzymes essential for maintaining the integrity of microorganisms’ cell walls.

These peptides can effectively impinge on a broad spectrum of microorganisms including fungi, bacteria, and viruses (Figure 1). Notably, endogenous AMPs are recognized for being highly selective against pathogens, and for the most part spares untransformed mammalian cells. Classical mechanisms which mediate their antibiotic actions include their penetration of the plasma membrane or cell wall, thus resulting in lysis or disruption of ionic gradients; binding to and damaging nucleic acids (e.g., DNA); and blockade of enzymes essential for maintaining the integrity of microorganisms’ cell walls (28–37) (Figure 2). Amphipathicity (i.e., the spatial segregation of cationic and hydrophobic residues) in particular appears to be a major determinant of function. Other physicochemical considerations that may impact structure–activity relationships include the amino acid sequence, net charge, hydrophobicity, structural folding (i.e., secondary structure, dynamics, and orientation) in membranes, oligomerization, peptide concentration, and membrane composition (38).

## Immunomodulatory Effects of AMPs

Innate immunological responses to infectious agents, including the secretion of AMPs, are foremost initiated and orchestrated by the precise interaction between pathogen-derived ligands and immune receptors. Besides their canonical functions in carrying out microbicidal actions, AMPs are increasingly recognized to interact with host cells to influence diverse signaling cascades which may enhance the resolution of infections. For instance, β-defensins, a peptide active against many Gram-negative and -positive bacteria and viruses, also serves as a ligand for the CCR6 chemokine receptor that is expressed on T lymphocytes and dendritic cells, hence serving as a bridge between the innate and adaptive arms of host immunity. Other hitherto-unappreciated consequences on host immune cells have been described, including altering host gene expression; inducing chemokine secretion; modulating the activation or death of neutrophils, T lymphocytes, and dendritic cells; regulating cellular differentiation pathways; and promoting immune-mediated wound healing (39–41).

The detailed spatiotemporal control of these interactions between AMPs and host cells are hitherto less well known. Several models have been proposed, including the “alternate ligand model,” wherein AMPs transduce intracellular signaling cascades by acting as ligands for cell surface receptors, and the “membrane disruption model,” in which AMPs focally modify a part of the receptor to alter their functions. Furthermore, the “trans-activation model” posits that the indirect action of AMPs may lead to the production of membrane-bound factors that are capable of inducing receptor activation. It has also been shown that the scavenging of the endotoxin lipopolysaccharide (LPS) by AMPs may impede LPS binding with toll-like receptor 4, thus suppressing a proinflammatory process (42).

## AMPs and Cancer Treatment

**Figure 3:**
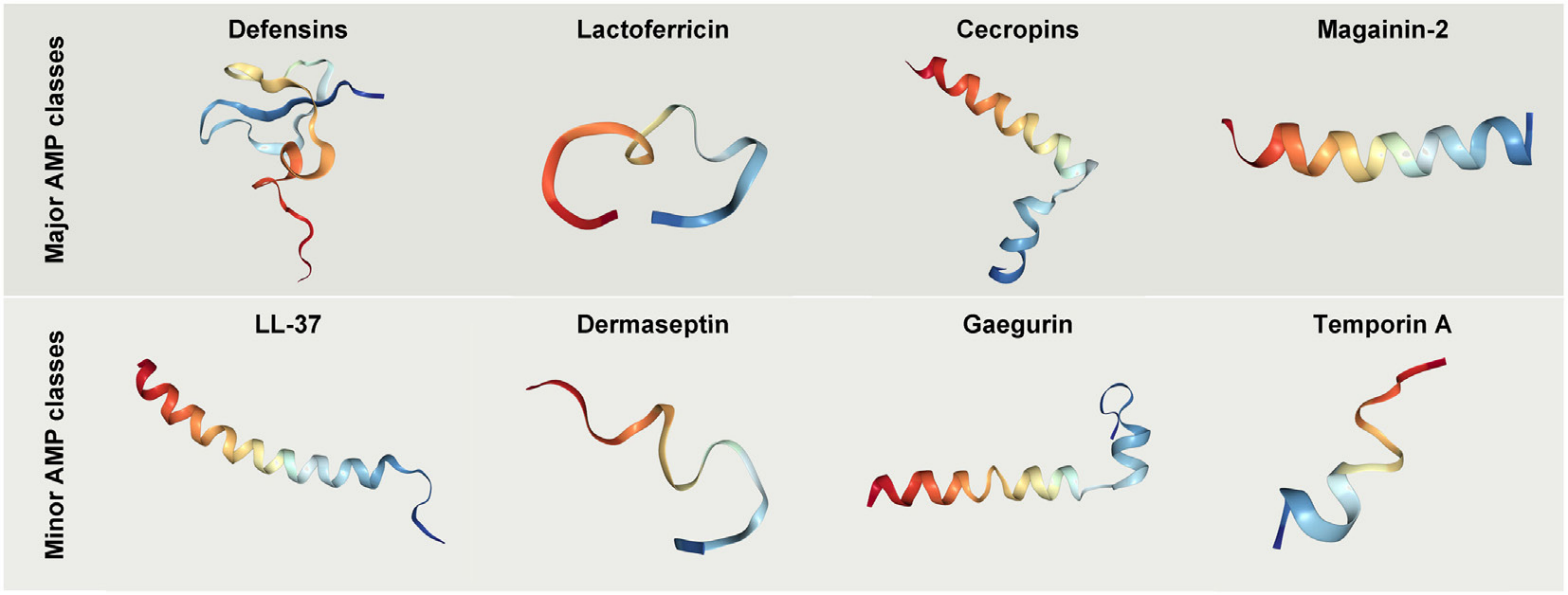
Crystal structures of selected antimicrobial peptides (AMPs) with anticancer potential. Protein databank accession codes: human beta-Defensin-3, IKJ6; cecropin B derivative CB1a, 2IGR; lactoferricin B, 1Y58; magainin 2, 2MAG; LL-37, 2K6O; Dermaseptin analog NC12-K4S4(1–13)a, 2DCX; Gaegurin 4, 2G9L; Temporin-1 Ta, 2MAA.

Henceforth in this review, we have defined classes of AMPs as “major” or “minor,” respectively, depending on whether their anticancer properties have been the subject of intensive study or are less well described (Figure 3).

## Major Therapeutically Relevant Classes of AMPs

### Defensins

**Figure 4:**
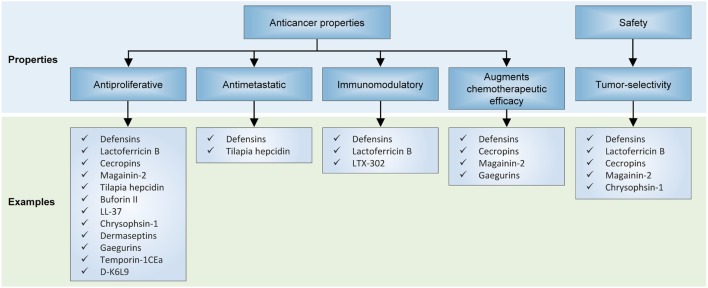
Effects of antimicrobial peptides on in preclinical models of neoplasia. Several recurrent themes have emerged from unfolding research on their anticancer properties, including their antiproliferative and antimetastatic capabilities, invigoration of antitumor immunity, activity against multidrug-resistant cancer cells, and selectivity for cancer cells but not normal cells.

The defensins are cationic peptides produced by eukaryotes and comprise two superfamilies which have undergone divergent evolution in terms of sequence, structure, and function. The anticancer properties of human defensins are featured in a rapidly growing body of findings, and encouraging preclinical results have been obtained with the treatment of cancer cells or xenograft models with various natural or synthetic defensins. For instance, it has been shown that natural human β-defensin-3 (hBD-3) is capable of suppressing VEGF-induced cancer cell migration capabilities (43–46). In another study, hBD-3 were shown to be produced by tumor-infiltrating monocytes and inhibited the invasiveness and motility of colon cancer cells in a dose-dependent and paracrine fashion (45). Considering that these *in vitro* malignant phenotypic traits correlate with a primary tumor’s propensity to establish life-threatening metastatic outgrowths, the finding that defensins are effective at repressing cancer cell motility suggests that they can be developed as potential antimetastatic agents (Figure 4).

Furthermore, natural defensins appear to exert antiproliferative and proapoptotic effects on cancer cells and to induce cell cycle arrest (44, 47–51), which are evidenced by increases in the levels of phosphorylated retinoblastoma protein, suppressed activities of transcriptional and cell cycle cyclin-dependent kinases and their catalytic cyclin partners (52), and enhanced expression of caspase 7 and 9 and other markers of apoptosis. Interestingly, human beta-defensin-2 (hBD-2) have been shown to also reduce the viability of melanoma cells through the downregulation of BRAF (52). Besides their natural derivatives, synthetic defensin analogs may be designed for greater anticancer efficacy: Du et al. demonstrated that recombinant tailored defensin (DF-HSA) comprising human β-defensin-2 (DF) and human serum albumin (HSA) was more effective than natural β-defensin at curbing the proliferation of K-Ras-mutant MIA PaCa-2 cells and suppressing the growth of a pancreatic carcinoma xenograft (53).

Two additional facets of human defensins warrant discussion: first, it is notable that they appear to have an impressive level of specificity for tumor cells, yet do not appear to exert palpable cytotoxic or cytostatic effects against normal untransformed cells (48, 50, 51, 54). It has been shown that defensins induce apoptosis in MCF-7 cells *via* the intrinsic pathway, enhanced MAPK p38 phosphorylation, as well as increased expression of cytochrome c, Apaf-1, caspase 7 and 9, but did not affect the membrane potential and calcium flow (48). Another study indicated that Laterosporulin10, a defensin-like anticancer bacteriocin, results in apoptotic and necrotic death of MCF-7, HEK293T, HT1080, HeLa, and H1299 cells (50).

This observation is arguably consistent with the fact that human AMPs are endogenously derived, and therefore are designed to avoid causing overt collateral toxicity to normal healthy tissues during an inflammatory response. Second, antimicrobial defensins may present novel opportunities to address unmet clinical issues such as chemotherapeutic resistance. For instance, defensins have been shown to potentiate cancer cell-kill in combination with cytotoxic chemotherapeutic agents such as doxorubicin in multidrug-resistant cancer cells (51, 54).

Another compelling application for defensins is their significant potential to augment the effectiveness of cancer immunotherapy. Li et al. for instance employed a recombinant plasmid which expresses beta-defensin 2 and evaluated its potential as both cancer gene therapy and immunotherapy (55). *In vitro* and *in vivo* results indicated that physiological changes occurred in immature dendritic cells in a fashion which is likely to enhance adaptive anticancer immunosurveillance (55).

### Lactoferricin B

Lactoferrin is an 80 kDa iron-sequestering glycoprotein present in exocrine secretions such as milk and in the granules of polymorphonuclear leukocytes, and represents another class of AMPs which have also been at the focal point of research into their anticancer properties. Lactoferricin B possess antitumor capabilities as it is capable of exerting lethal, selective destabilizing effects on cancer cell cytoplasmic and mitochondrial membranes (11), and has been shown to be effective against colorectal, neuroblastoma, and melanoma cancer cells (11, 56). As alluded to earlier in the article, AMPs may assume various conformations. A recent study demonstrated that bovine lactoferrin (bLf), cyclic LfcinB, and linear LfcinB were all capable of activating multiple antineoplastic signaling cascades, including p53 induction, apoptosis, and anti-angiogenic pathways as revealed by transcriptomic analyses (57). Furthermore, both bLf and LfcinB led to the induction of proapoptotic pathways mediated by caspase-8, p53, and p21 in colorectal carcinoma cells (57). Like the defensin proteins, lactoferricin appears to have immunomodulatory effects, and have been shown to orient lymphocytes toward eradicating cancer cells (58).

### Cecropins A and B

Cecropins represent a class of small and basic peptides prototypically ranging between 31 and 39 amino acid residues with a strongly basic N-terminus and hydrophobic C-terminus and were initially isolated from the silk moth *Hyalophora cecropia*. Again as with a recurring theme regarding AMPs, cecropins A and B have been shown to have selective cytotoxic and cytostatic effects on bladder neoplasms but not on human fibroblast cell lines (59), while ABP-dHC-Cecropin A and its analog ABP-dHC-Cecropin A-K are cytotoxic against leukemic but not non-cancerous cell lines (60). Cecropin A has been shown to induce apoptosis in the promyelocytic cell line HL-60 through a ROS signaling mechanism (61), and in human hepatocellular carcinoma cells *via* expression of Fas, Fas-L, caspase-3, and -8 (12). CecropinXJ induces growth inhibition, S-phase arrest and apoptosis in hepatocellular carcinoma cells through expression of caspase-3 and poly(ADP-ribose) polymerase, and downregulation of B cell lymphoma 2 (Bcl-2) (62). One particularly enticing finding has been the result that *in vitro*, cecropin A enhances the anticancer effects of common chemotherapeutic agents against squamous skin cancer cells, which could open up new possibilities for rational combination strategies consisting of these two modalities (63).

### Magainin II (MG2)

Magainin II (MGN-II) is an AMP isolated from the skin of the African clawed frog *Xenopus laevis* which has demonstrated potent anticancer effects in various hematopoietic and solid malignancies. As an ionophoric peptide with a helical structure, it has been shown to perforate cancer cell membranes to act as ion channels, causing cytolysis of cancerous cells (64). MGN-II greatly enhances the tumoricidal effects of cytotoxic chemotherapeutic agents. For instance, magainin A (MAG A) and magainin G (MAG G) have been shown to have synergistic effects when used with chemotherapy against non-small cell lung cancer cell lines (65). MGN-II also displays tumor-selectivity; for instance, they have been demonstrated to lyse various hematopoietic tumor and solid tumor cells but have little or no effect on normal human fibroblasts and peripheral blood lymphocytes (8, 10, 12).

Derivatives of MGN-II have also been synthesized to enhance their cancer-specific cytotoxic properties (18, 64, 66). For instance, a magainin II-bombesin conjugate (MG2B) has demonstrated enhanced activity at a lower dose against a wide range of human cancer cells, without adverse effects on normal cells, as well as potent antitumor activity in a murine model of breast cancer (66). In yet another example, the fusion peptide MG2A, which was synthesized by conjugating MGN-II to the NH2-terminal of the cell-penetrating peptide penetratin (Antp), exhibited augmented cytotoxicity against a variety of human cancer cells and rat glioma cells, while having very limited off-target effects on normal cells (18).

## Minor Therapeutic Classes of AMPs

Structurally, LTX-302 is a 9-mer peptide derived from its parental peptide LfcinB, and features an α-helical secondary structure optimized to exert greater antitumor activity (67–69). The effects of LTX-302 have been examined *in vivo* against A20 B cell lymphomas in BALB/c mice (69). Interestingly, antitumor activity hinged on the mobilization and activation of CD4 and CD8 T-lymphocytes, but ultimately LTX-302 administration induced long-lived cellular immunity directed against lymphoma cells and mediated complete regression of tumors in the majority of xenograft mice (69).

Tilapia hepcidin (TH) 1–5 represent three hepcidin-like AMPs (TH1–5, TH2–2, and TH2–3) extracted from tilapia (*Oreochromis mossambicus*). They were previously shown by Chen et al. to specifically inhibit the growth of human fibrosarcoma HT1080 cells *via* cell membrane-perforating mechanisms, and to also diminish cell migration capabilities (10). These findings were corroborated by Chang et al. who found that TH1–5 decreased colony formation and induced rapid cell death in various human cancer cell lines (70).

Buforin II is a 21-residue α-helical AMP with sequence similarity to the N-terminal region of histone H2A. Buforin IIb is a synthetically derived analog of buforin II, modified to contain a proline residue in between two α-helices (71). Buforin IIb was found in a study to have activity against breast cancer cells MX-1 and MCF-7, and to suppress tumor growth in a mouse xenograft through anti-vasculogenic and anti-angiogenic mechanisms (13). It is postulated that the glycosylation of breast cancer cells plays a significant role in enabling interaction with this AMP, thereby allowing it to exert anticancer effects (13).

LL-37 is a derivative of human cathelicidin-derived α-helical AMPs. Its precursor hCAP18 is found in body fluids and functions as a peptide antibiotic and signaling molecule (72). In a prior study, human colon cancer cells (HCT116 and LoVo) treated with FK-16, a 16-residue fragment of LL-37 underwent caspase-independent apoptosis and autophagy as a result of activation of the p53-Bcl-2/Bax cascade (16).

Chrysophsin-1 is an α-helical AMP found in the gill cells of red sea bream, which is distinct due to its hemolytic properties, in addition its antibiotic functions (73). In 2011, Hsu et al. tested the effects of chrysophsin-1 on a wide panel of cancer cell lines (74), and showed that low-dose chrysophsin-1 selectively culls tumor cells *via* a membrane-depolarizing lytic mechanism (74).

Dermaseptins B1–B6 are six related peptides of 24–33 residues in length which are constitutively expressed in the skin secretions of the South American frog *Phyllomedusa bicolor*. Zoggel and colleagues demonstrated that the B2 peptide had antiproliferative and anti-colony-forming effects on a wide range of human cancer cells as well as xenograft model of prostate adenocarcinoma (15).

Gaegurins are a family of six AMPs isolated from *Rana rugosa* and are broadly divided into two subfamilies based on their length and sequence. The family II peptide GGN6, as well as its derivative PTP7, has been shown in a previous experiment by Kim et al. to have potent anticancer effects against multiple human cancer cell lines, including A549, PC-3, and MCF-7 (17). Intriguingly, as has also been demonstrated in aforementioned AMPs, GGN6 derivatives potently induced cell cycle arrest and apoptosis in multidrug-resistant cancer cells, suggesting its potential to be deployed in the treatment of refractory malignancies (17).

Polybia-MPI is a cationic peptide extracted from the social wasp *Polybia paulista*. Polybia-MPI is characterized by potent bactericidal (against both Gram-positive and Gram-negative bacteria), fungicidal, and tumoricidal biochemical properties, while being relatively non-toxic to human red blood cells and normal fibroblasts (75, 76). Polybia-MPI and its derivatives have been shown to induce pore formation leading to the death of prostate cancer, bladder cancer, and drug-resistant myelogenous leukemic cells (75, 76).

Temporin-1CEa is an amphipathic AMP secreted by the skin of the Chinese brown frog *Rana chensinensis*. Studies have demonstrated that the treatment of a range of human cancer cell lines with temporin-1CEa rapidly induces tumor cell death by disrupting their cell membrane and mitochondria (77, 78). Melanoma cells seem particularly susceptible, perhaps because of their overexpression of phosphatidylserine, which has high affinity for temporin-1CEa (78).

D-K6L9 is a peptide is bound by phosphatidylserine which is capable of inducing tumor necrosis. Its administration to B16-F10 murine melanoma tumors inhibited its growth, whereas therapeutic cessation led to tumor relapse (79). Combinations comprising D-K6L9 with glycyrrhizin (an inhibitor of HMGB1 protein), BP1 peptide, and interleukin (IL)-12 exhibit antitumor efficacy. When glycyrrhizin or BP1 is combined with D-K6L9, the growth of tumors was suppressed during the period of their administration. Long-lasting tumor growth suppression effect was achieved by combining D-K6L9 plus IL-12. Two months after therapeutic cessation, a remarkable 60% of animals remained alive. Significantly prolonged survival was observed in both mice bearing B16-F10 tumors as well as in mice bearing C26 colon carcinoma tumors (79).

## AMPs in Clinical Trials

Some cationic AMPs may exert their microbicidal effects chiefly through the potent induction of host immunoreactivity, rather than through direct modes of action. It is for this reason that their potential for use as adjuncts to current anticancer modalities has galvanized significant interest over the past few years, boosted by tantalizing efficacy results from recent clinical trials of cancer immunotherapies.

The safety profile of AMPs deserves mention, if only briefly. Whereas in the context of oncologic indications, these biologics remain experimental (Table 1), it should be noted that several AMPs have transitioned to phase II clinical trial evaluation or have even obtained U.S. Food and Drug Administration approval for use in the treatment of various infectious diseases (80, 81), such as pexiganan acetate (MSI-78) (82), hLF1–11 (83), omiganan (MBI-226) (81), CZEN-002, and novexatin (NP-213) (84) (Table 2).

**Table 1:** Antimicrobial peptides for oncologic indications in ongoing clinical trials.

Phase	Peptide name	Identifier number	Condition	Administration route
I	LL37	NCT02225366	Metastatic melanoma	Intratumoral
	GRN-1201	NCT02696356	Solid tumors	Intravenous
	LTX-315	NCT01058616	Solid tumors	Intravenous
	WT-2725	NCT01621542	Hematological malignancy and solid tumors	Intravenous

II	SGX942	NCT02013050	Head and neck cancer	Intravenous
	ANG-1005	NCT02048059	Breast and brain metastasis	Intravenous

III	ITK-1	UMIN000011308	Glioblastoma and prostate cancer	Intravenous

**Table 2:** Antimicrobial peptides for infectious diseases indications in ongoing clinical trials.

Phase	Peptide name	Identifier numbers	Condition	Administration route
II	NP213 (Novexatin)	NCT02933879	Fungal nail infection	Topical
	PAC-113	NCT00659971	Oral candidiasis in HIV patients	Topical
	MBI-226	NCT00211523	Acne vulgaris and acne	Topical
	Dalbavancin	NCT02685033	Acute hematogenous osteomyelitis	Intravenous
	Brilacidin	NCT02052388	Skin and bacterial infection	Topical
	CLS001 (Omiganan)	NCT02596074	Acne vulgaris	Topical

III	Pexiganan (MSI-78)	NCT01594762	Diabetic foot infection	Topical
	Surotomycin	NCT01597505	Clostridium difficile associated diarrhea	Oral
	CLS001 (Omiganan)	NCT02576847	Papulopustular rosacea	Topical
	P2TA	NCT01417780	Necrotizing soft tissue infection	Intravenous

The first AMP developed commercially was pexiganan acetate (MSI-78) (82). A number of AMPs are being developed for systemic applications. For instance, hLF1-11, a cationic fragment comprised N-terminal amino acids 1–11 of human lactoferricin, is intravenously administered for the treatment of severe bacterial and fungal infections in immunosuppressed stem cell transplant recipients (83). Another AMP that is at an advanced stage of clinical trialing is omiganan (MBI-226), a derivative of indolicidin which was purified from bovine neutrophils, being tested as a topical gel for the prophylaxis of contamination of central venous catheters (81). Additional examples abound: Fopical pexiganan might be an effective alternative to oral antibiotic therapy compared to ofloxacin for treatment of mildly infected foot ulcer in diabetic patients (85). Novexatin (NP-213), a cyclic and highly cationic peptide based on human α- and β-defensins, has shown promise in treating recalcitrant fungal infections in toenails while CZEN-002, a dimeric peptide sequentially derived from α-melanocyte-stimulating hormone, is targeting vaginal candidiasis (84).

Antimicrobial peptides are only beginning to encroach into the oncological sphere, and therefore efficacy data are relatively limited (Table 1). However, the safety data in infectious diseases trials, albeit indirectly, substantiate the notion that AMPs could also be well tolerated in cancer patients. An example of an ongoing oncology trial is NCT02225366, wherein the optimal biological dose and therapeutic activity of LL37 against metastatic melanoma is being evaluated in a phase I setting. LL37 is being administered intratumorally in patients with documented metastatic melanoma and at least three cutaneous lesions measuring with stage IIIB, IIIC, or IV or nodal lesions.

## Conclusion and Future Directions

Therapeutic resistance and metastatic dissemination are some of the most clinically challenging aspects of cancer. There is now an abundance of evidence that AMPs hold substantial potential for filling these therapeutic voids in clinical oncology. In this review, we have discussed evidence for their antiproliferative, proapoptotic, and antimetastatic effects on cancer cells. Crucially, AMPs have also shown activity against multidrug-resistant cancer cell lines, and therefore could prove valuable for the treatment of advanced, refractory cancers. Hence, we envision that in the future, combination strategies involving this novel therapeutic class with conventional cancer treatments (targeted therapies, immunotherapies, and chemotherapy) may improve treatment outcomes.

**Figure 5:**
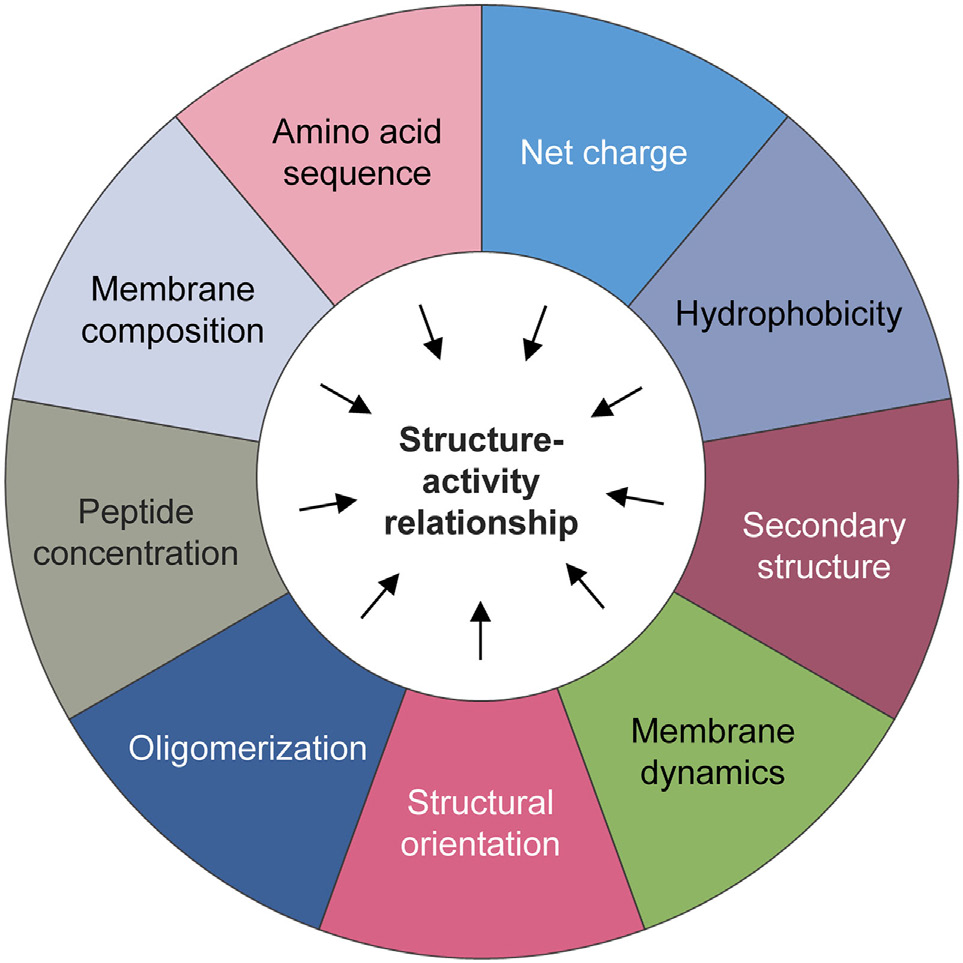
Physicochemical considerations that may impact structure–activity. A number of biochemical factors need to be considered when predicting the anticancer activity of natural or synthetic antimicrobial peptides, including but not limited to the amino acid sequence, net charge, hydrophobicity, structural folding (i.e., secondary structure, dynamics, and orientation) in membranes, oligomerization, peptide concentration, and membrane composition.

Nevertheless, significant challenges lie ahead in the path toward their clinical development and deployment. Toxicity continues to feature as a prominent concern, especially with regardsto the administration of non-human natural or synthetic AMPs. However, lessons can be learnt from the field of infectious diseases, where several AMPs have transitioned to clinical trials or have even gained a foothold in clinical care. One area in which the therapeutic index of these peptides can be improved is through the development of innovative formulations and drug delivery systems. Structure–activity relationship, lead identification, and optimization studies are also crucial to improving the anticancer efficacy of AMPs (Figure 5). Yet, these will also require greater understanding of their underlying anti-tumorigenic mechanisms, which hitherto remain somewhat speculative. It is also worth noting that the vast majority of AMPs that exist in nature have yet to be characterized, and given their potential for therapeutic applications, there are certainly compelling reasons to conduct more comprehensive surveys to identify drug candidates. However, with focused efforts to overcome these limitations and obstacles, and a rigorous commitment to translate—at a reasonable cost—these promising peptides into cancer care, the future for this emerging therapeutic class looks exceptionally bright.

## Author Contributions

All authors listed have made a substantial, direct, and intellectual contribution to the work and approved it for publication.

## Conflict of Interest Statement

The authors declare that the research was conducted in the absence of any commercial or financial relationships that could be construed as a potential conflict of interest.
